# Genetic analysis and prenatal diagnosis of 20 Chinese families with oculocutaneous albinism

**DOI:** 10.1002/jcla.23647

**Published:** 2020-10-30

**Authors:** Chenyang Xu, Yanbao Xiang, Huanzheng Li, Yunzhi Xu, Yijian Mao, Lili Zhou, Xueqin Xu, Shaohua Tang

**Affiliations:** ^1^ Key Laboratory of Birth Defects Department of Genetics Wenzhou Central Hospital Wenzhou China; ^2^ Key Laboratory of Medical Genetic School of Laboratory Medicine and Life Science Wenzhou Medical University Wenzhou China

**Keywords:** *HPS1*, OCA, prenatal diagnosis, *TYR*, whole exome sequencing

## Abstract

**Background:**

Oculocutaneous albinism (OCA) is a group of heterogeneous genetic disorders characterized by abnormal melanin synthesis in the hair, skin, and eyes. OCA exhibits obvious genetic and phenotypic heterogeneity. Molecular diagnosis of causal genes can be of help in the classification of OCA subtypes and the study of OCA pathogenesis．

**Methods:**

In this study, Sanger sequencing and whole exome sequencing were used to genetically diagnose 20 nonconsanguineous Chinese OCA patients. In addition, prenatal diagnosis was provided to six OCA families.

**Results:**

Variants of *TYR*, *OCA2*, and *HPS1* were detected in 85%, 10%, and 5% of affected patients, respectively. A total of 21 distinct variants of these three genes were identified. Exons 1 and 2 were the hotspot regions of the *TYR* variants, and c.895C > A and c.896G > A were the hotspot variants. We also found seven novel variants: c.731G > A, c.741C > A, c.867C > A, and c.1037‐2A > T in *TYR*, c.695dupT and c.1054A > G in *OCA2*, and c.9C > A in *HPS1*. Genetic tests on six fetuses revealed three carrier fetuses, two normal fetuses, and one affected fetus. The follow‐up results after birth were consistent with the results of prenatal diagnosis (one fetus terminated during pregnancy was not followed up).

**Conclusions:**

This study expands our understanding of the genotypic spectrum of the Chinese OCA population. The findings indicate that prenatal diagnosis can provide important information for genetic counseling.

## Graphical abstract

We showed the clinical phenotype and genetic diagnosis results of 20 OCA patients. A total of 21 distinct alleles, including seven novel variants, of the *TYR*, *OCA2*, and *HPS1* were detected by Sanger sequencing or whole exome sequencing. This study expands our understanding of the genotypic spectrum of the Chinese OCA population and highlights that whole exome sequencing can contribute to more accurate and efficient clinical diagnoses.

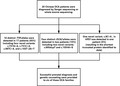

## 1 INTRODUCTION

Albinism is a group of heterogeneous genetic disorders characterized by complete absence or reduction of melanin biosynthesis. Albinism can be categorized as either oculocutaneous albinism (OCA) or ocular albinism (OA). The main clinical features of OCA are hypopigmentation of the skin, hair, and eyes, whereas OA only affects the eyes. Ocular characteristics include reduced iris pigment, reduction in visual acuity, nystagmus, strabismus, and photophobia. OCA is the most common form of albinism, affecting about 1 in 20 000 individuals worldwide.^1^ The prevalence is higher (about 1 in 18 000) in the Chinese population.^2^

Based on the complexity of the system involved, OCA can be further classified as either nonsyndromic OCA or syndromic OCA. OCA1 (OCA1A, OMIM 203100; OCA1B, OMIM 606952), OCA2 (OMIM 203200), and OCA3 (OMIM 203290) are the most common subtypes of nonsyndromic OCA, caused by homozygous or compound heterozygous variants in the tyrosinase gene (*TYR*, OMIM 606933), OCA2 melanosomal transmembrane protein gene (*OCA2*, OMIM 611409), and tyrosinase‐related protein 1 gene (*TYRP1*, OMIM 115501), respectively.^3^, ^4^ In addition, four rare subtypes of OCA, OCA4 (OMIM 606574), OCA5 (OMIM 615312), OCA6 (OMIM 113750), and OCA7 (OMIM 615179) have also been identified.^5^ Recently, pathogenetic variations in the solute carrier family 45 member 2 gene (*SLC45A2*, OMIM 606202), solute carrier family 24 member 5 gene (*SLC24A5*, OMIM 609802), and leucine‐rich melanocyte differentiation‐associated protein gene (*LRMDA*, OMIM 614537) have been found to be responsible for OCA4, OCA6, and OCA7. Syndromic OCA, such as Chediak‐Higashi syndrome (CHS, OMIM 214500), Hermansky‐Pudlak syndrome (HPS, OMIM 203300), and Griscelli syndrome (GS, OMIM 214450), can also present with platelet storage pool deficiency or immunodeficiency.^6^, ^7^ Several genes, including HPS1 biogenesis of lysosomal organelles complex 3 subunit 1 gene (*HPS1*, OMIM 604982), lysosomal trafficking regulator gene (*LYST*, OMIM 606897), and myosin VA gene (*MYO5A*, OMIM 160777), are considered as the main candidate genes involved in the pathogenesis of syndromic OCA.

Due to the genetic heterogeneity of OCA and the overlapping phenotypes of different OCA subtypes, it is difficult to distinguish between different OCA subtypes and to identify causal genes. As such, molecular genetic testing has become the only reliable method for OCA classification.^8^ In this study, Sanger sequencing and whole exome sequencing (WES) were used for pathogenic variant screening of OCA‐causing genes in 20 Chinese families with albinism. In addition, prenatal diagnosis and informative genetic counseling were provided to six OCA families.

## 2 MATERIALS AND METHODS

### 2.1 Patients

The present study was approved by the institutional research ethics committee of Wenzhou Central Hospital, Zhejiang, China, and the study protocol conforms to the ethical guidelines of the Declaration of Helsinki. Written informed consent was obtained from the patient or the guardians of the patient. A total of 20 OCA families from East China were screened between 2013 and 2019, including six families who underwent prenatal diagnosis. All patients denied any family history of consanguinity. The following clinical data were collected: skin, hair, and iris color; ophthalmological findings (photophobia, nystagmus, and eyesight); and other phenotypes (eg, abnormalities of the immune system, hemorrhagic diathesis).

### 2.2 Sanger sequencing

The genomic DNA (gDNA) was extracted from amniocytes or peripheral blood using a Qiagen DNA Blood Midi/Mini kit (Qiagen), following the manufacturer's protocol. Variant screening of the *TYR* gene in 12 families (family 1‐10, 19, and 20) was performed with direct Sanger sequencing. Polymerase chain reaction (PCR) primers were designed by Primer Premier version 5.0 and contained the entire coding regions and the flanking introns of the *TYR* gene (Table S1). The 20 μL PCR reaction mixture contained 10‐50 ng template DNA, 10 μL Premix EX Taq HS (Takara), and 1 μL of each primer. Touchdown PCR was performed as follows: 95°C for 15 minutes; 11 cycles of 95°C for 45 seconds, 60°C‐0.5°C for 45 seconds, 72°C for 45 seconds; 24 cycles of 95°C for 45 s, 54°C for 45 seconds, 72°C for 45 seconds; and 72°C for 7 minutes. The PCR products were sequenced using an ABI 3130 automated DNA sequencer (Applied Biosystems). DNASTAR Lasergene SeqMan software was used for DNA sequence assembly, and sequences were compared with a wild‐type reference sequence.

### 2.3 Whole exome sequencing

WES was performed in the other 10 families (family 11‐20); this included two families (family 19 and 20) in which direct Sanger sequencing failed to identify any pathogenic variants. Exon‐containing fragments were enriched by SureSelect Human All Exon V6 (Agilent, USA), and a HiSeq2500 sequencer was used for sample sequencing (Illumina). Paired‐end sequencing was performed for each sample, and more than 95% of bases in the targeted regions were covered by at least 20 reads. Raw data generated by WES were filtered to obtain high‐quality clean reads and were further aligned to the NCBI Human Reference Genome (hg19/GRCh37) using the Burrows‐Wheeler Aligner (BWA). Then, SAMtools and Genome Analysis ToolKit (GATK) were used to detect single nucleotide polymorphisms or sequence variants in BAM files. Variants located in coding regions or exon‐intron junctions of 33 candidate genes (Table S2) responsible for albinism or diseases whose phenotypes partially overlap with albinism were analyzed emphatically.

### 2.4 Variant analysis

Data from WES or Sanger sequencing were analyzed in accordance with the following criteria. First, variants with a minor allele frequency > 0.01 in Single Nucleotide Polymorphism database (dbSNP, http://www.ncbi.nlm.nih.gov/snp), 1000 Genomes Project (http://browser.1000genomes.org), genome Aggregation Database (gnomAD, http://gnomad.broadinstitute.org/), Trans‐Omics for Precision Medicine Program (TOPMed, https://www.nhlbi.nih.gov/science/trans‐omics‐precision‐medicine‐topmed‐program), and Exome Aggregation Consortium database (ExAC, http://exac.broadinstitute.org/) were filtered out. Second, databases such as the Albinism Database (http://www.ifpcs.org/albinism), ClinVar (http://www.ncbi.nlm.nih.gov/clinvar), and the Human Gene Mutation Database (HGMD, http://www.hgmd.org) were used to determine the pathogenicity of variants. Third, all whole genome variants were subjected to biological effects analysis, which included the use of programs such as MutationTaster (http://www.mutationtaster.org), Polymorphism Phenotyping v2 (PolyPhen2, http://genetics.bwh.harvard.edu/pph2), Protein Variation Effect Analyzer (PROVEAN, http://provean.jcvi.org/index.php), Sorting Intolerant From Tolerant (SIFT, http://sift.jcvi.org), and Human Splicing Finder (HSF, http://www.umd.be/HSF) to predict whether an amino acid substitution or indel was more likely to be pathogenic. Finally, all detected variants were classified as pathogenic, likely pathogenic, uncertain significance, likely benign, or benign, according to the American College of Medical Genetics and Genomics (ACMG) guidelines.^9^

### 2.5 Pedigree analysis and prenatal testing

All detected (likely) pathogenic variants were subsequently tested on parents or other available family members. The relevant sequence variants identified by WES were verified by Sanger sequencing, as described above. Six of the 20 families underwent amniocentesis between 18‐ and 24‐week gestation for prenatal diagnosis. Fetal gDNA was extracted using a QIAamp DNA Mini Kit (Qiagen), following the manufacturer's protocol; DNA was genotyped by Sanger sequencing. Monitoring for maternal contamination was performed with the analysis of 16 short tandem repeat (STR) loci in the fetal versus maternal gDNA using an STR Identifiler PCR Amplification Kit (Genesky Biotechnologies Inc).

## 3 RESULTS

### 3.1 Clinical phenotype

In this study, all patients had typical OCA symptoms of the skin, hair, and eyes. Clinical features of these 20 patients are described in Table 1. Among these patients, there were several special cases. Specifically, patient 10 died 15 days after birth due to congenital diaphragmatic hernia. In addition, patients 9, 19, and 20 had pigmented nevi on their bodies.

**TABLE 1:** Clinical manifestations and genotypes of the 20 OCA patients

Patient	Sex	Age (y)	Clinical features						Gene	Genotype			
			Skin color	Hair color	Iris color	Photophobia	Nystagmus	Myopia		Allele 1	FM	Allele 2	FM
1	M	12	White	White	Red brown	Positive	Positive	Severe	*TYR*	c.895C > A (p.R299S)	Pa	c.896G > A (p.R299H)	Ma
2	M	16	White	White	Brown	Negative	Negative	Mild	*TYR*	c.895C > A (p.R299S)	Pa	c.229C > T (p.R77W)	Ma
3	F	9	White	White	Brown	Positive	Positive	Moderate	*TYR*	c.867C > A (p.C289*)^a^	Pa	c.929dupC (p.R311Kfs*7)	Ma
4	M	25	White	White	Brown	Positive	Positive	Moderate	*TYR*	c.895C > A (p.R299S)	Pa	c.230_232dupGGG (p.E78_S79insG)	Ma
5	F	10	White	White	Brown	Positive	Positive	Severe	*TYR*	c.832C > T (p.R278*)	Pa	c.896G > A (p.R299H)	Ma
6	M	13	White	White	Red brown	Positive	Positive	Severe	*TYR*	c.731G > A (p.C244Y)^a^	Pa	c.832C > T (p.R278*)	Ma
7	M	13	White	White	Light yellow	Positive	Positive	Severe	*TYR*	c.929dupC (p.R311Kfs*7)	Pa	c.895C > A (p.R299S)	Ma
8	M	46	White	White	Brown	Positive	Positive	Severe	*TYR*	c.731G > A (p.C244Y)^a^	NA	c.895C > A (p.R299S)	Daughter
9	M	30	White	White	Red brown	Positive	Positive	Mild	*TYR*	c.1037‐2A > T^a^	Sister, affecting brother	c.346C > T (p.R116*)	Affecting brother
10	M	<1	White	Light yellow	Gray	NA	NA	NA	*TYR*	c.230_232dupGGG (p.E78_S79insG)	Pa	c.895C > A (p.R299S)	Ma
11	F	70	White	White	Red brown	Positive	Positive	Severe	*TYR*	c.164G > A (p.C55Y)	NA	c.164G > A (p.C55Y)	NA
12	M	9	White	White	Red brown	Positive	Positive	Severe	*TYR*	c.1199G > T (p.W400L)	Pa	c.1199G > T (p.W400L)	Ma
13	F	30	Pinkish White	Brown	Light brown	Negative	Negative	Moderate	*TYR*	c.325G > A (p.G109R)	NA	c.741C > A (p.C247*)^a^	NA
14	F	33	White	White	Red brown	Positive	Positive	Severe	*TYR*	c.896G > A (p.R299H)	Pa	c.896G > A (p.R299H)	Ma
15	M	38	White	White	Brown	Positive	Negative	Moderate	*TYR*	c.896G > A (p.R299H)	NA	c.896G > A (p.R299H)	NA
16	M	32	White	White	Brown	Positive	Positive	Mild	*TYR*	c.164G > A (p.C55Y)	NA	c.706T > C (p.W236R)	NA
17	M	26	Pinkish White	White	Brown	Negative	Positive	Mild	*TYR*	c.1204C > T (p.R402*)	NA	c.929dupC (p.R311Kfs*7)	NA
18	M	7	White	Golden	Gray blue	Positive	Positive	Mild	*OCA2*	c.1255C > T (p.R419W)	Pa	c.695dupT (p.A233Gfs*26)^a^	Ma
19	F	52	White	White	Blue	Positive	Negative	Mild	*OCA2*	c.1426A > G (p.N476D)	NA	c.1054A > G (p.R352G)^a^	Son
20	F	30	White	Brown	Brown	Positive	Positive	Severe	*HPS1*	c.9C > A (p.C3*)^a^	NA	c.9C > A (p.C3*)^a^	NA

Note

The sign "a" represents novel variants.

Abbreviations: F, female; FM, family member; M, male; Ma, maternal; NA, not available; Pa, paternal.

### 3.2 Identification of sequence variants

In the current study, (likely) pathogenic variants in the *TYR* (GenBank: NM_000372.5), *OCA2* (GenBank: NM_000275.3), and *HPS1* (GenBank: NM_000195.5) genes were identified in 85% (17/20), 10% (2/20), and 5% (1/20) of OCA patients, respectively. All identified variants are shown in Table 1 and Table 2.

**TABLE 2:** Variants detected in this study

Gene	Exon	Variant	Type	Frequency
*TYR*	EX1	c.164G > A (p.C55Y)	Missense	3/34
	EX1	c.229C > T (p.R77W)	Missense	1/34
	EX1	c.230_232dupGGG (p.E78_S79insG)	In‐frame	2/34
	EX1	c.325G > A (p.G109R)	Missense	1/34
	EX1	c.346C > T (p.R116*)	Nonsense	1/34
	EX1	c.706T > C (p.W236R)	Missense	1/34
	EX1	c.731G > A (p.C244Y)	Missense	2/34
	EX1	c.741C > A (p.C247*)	Nonsense	1/34
	EX2	c.832C > T (p.R278*)	Nonsense	2/34
	EX2	c.867C > A (p.C289*)	Nonsense	1/34
	EX2	c.895C > A (p.R299S)	Missense	6/34
	EX2	c.896G > A (p.R299H)	Missense	6/34
	EX2	c.929dupC (p.R311Kfs*7)	Frameshift	3/34
	IVS2	c.1037‐2A > T	Splicing	1/34
	EX4	c.1199G > T (p.W400L)	Missense	2/34
	EX4	c.1204C > T (p.R402*)	Nonsense	1/34
*OCA2*	EX7	c.695dupT (p.A233Gfs*26)	Frameshift	1/4
	EX10	c.1054A > G (p.R352G)	Missense	1/4
	EX13	c.1255C > T (p.R419W)	Missense	1/4
	EX14	c.1426A > G (p.N476D)	Missense	1/4
*HPS1*	EX3	c.9C > A (p.C3*)	Nonsense	2/2

Among the 17 patients affected by *TYR* variants, four were homozygous (patient 11, 12, 14, and 15) and the other 13 were compound heterozygous. Gene analysis revealed 16 distinct *TYR* alleles, including eight missense types, five nonsense types, one frameshift type, one in‐frame type, and one splice site variant; most were located in exon 1 or 2 (13/16). c.895C > A and c.896G > A were the highest frequency variations identified, followed by c.164G > A and c.929dupC; these four variants accounted for 52.9% (18/34) of the total *TYR* variants. In addition, four novel variants were detected: c.731G > A, c.741C > A, c.867C > A, and c.1037‐2A > T.

Two patients were found to carry four distinct *OCA2* alleles. Patient 18 was compound heterozygous for c.695dupT (p.A233Gfs*26), a novel frameshift insertion, and c.1255C > T (p.R419W). We also found that c.695dupT was a maternal variant, and c.1255C > T was a paternal variant. Patient 19 was compound heterozygous for the novel missense variant c.1054A > G (p.R352G) and c.1426A > G (p.N476D).

Patient 20 was homozygous for the c.9C > A (p.C3*) variant in *HPS1*. This novel nonsense variant was located in exon 1 and predicted to result in the shortest truncated protein, only containing three residues.

### 3.3 Pathogenicity analysis of novel variants

**FIGURE 1:**
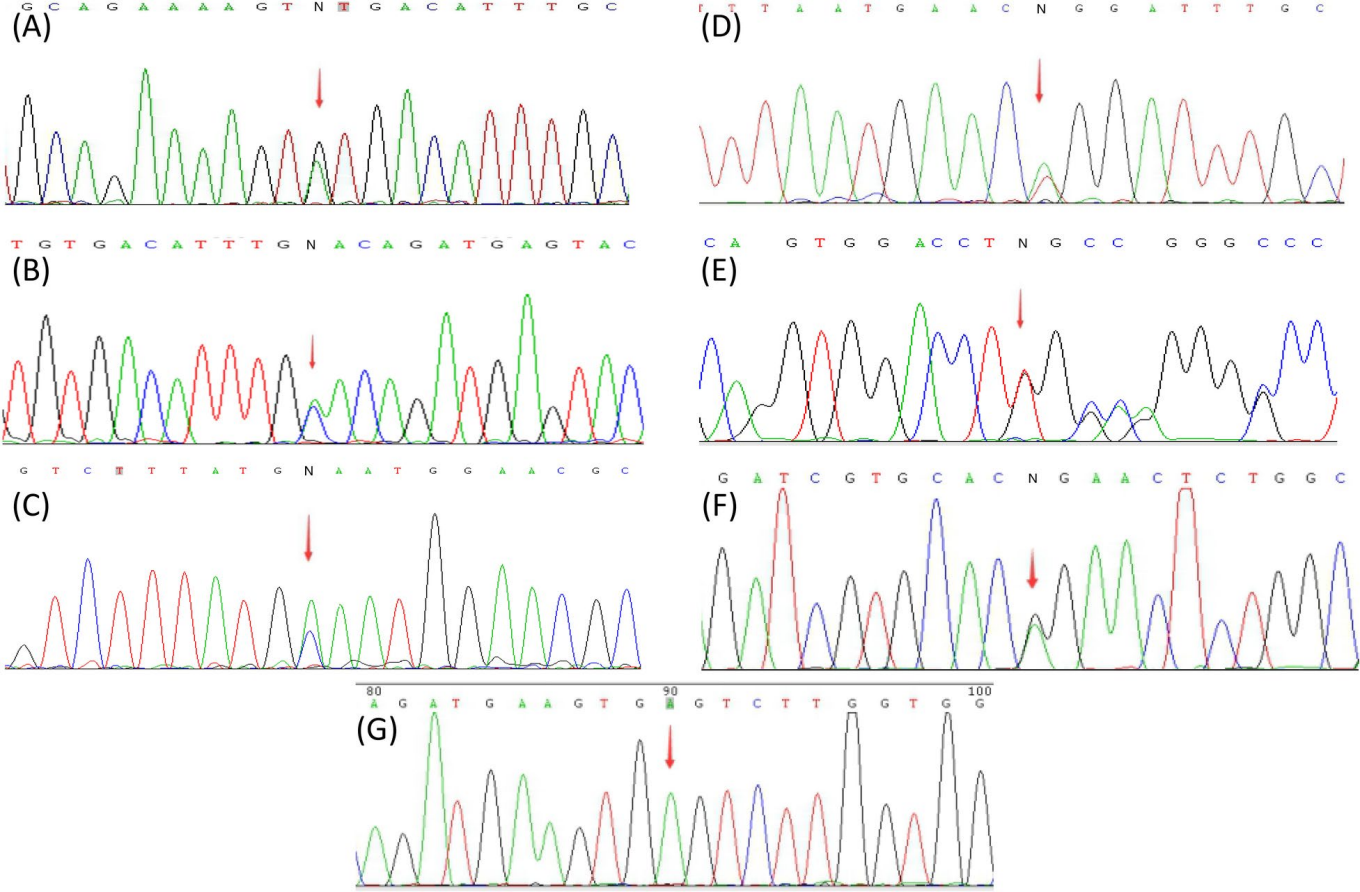
Sanger sequencing result of seven novel variants. A, Heterozygous for c.731G > A in *TYR*; (B) heterozygous for c.741C > A in *TYR*; (C) heterozygous for c.867C > A in *TYR*; (D) heterozygous for c.1037‐2A > T in *TYR*; (E) heterozygous for c.695dupT in *OCA2*; (F) heterozygous for c.1054A > G in *OCA2*; (G) homozygous for c.9C > A in *HPS1*

Seven novel variants were identified in this study, including c.731G > A, c.741C > A, c.867C > A, and c.1037‐2A > T of the *TYR* gene, c.695dupT and c.1054A > G of the *OCA2* gene, and c.9C > A of the *HPS1* gene (Figure 1 and Table 3). None of these were present in the ClinVar, HGMD, or Albinism databases. Almost all of these seven variants were absent from the 1000 Genomes Project, gnomAD database, TOPMed, and ExAC, except for c.731G > A and c.741C > A, which were present at extremely low frequencies in TOPMed and gnomAD, respectively. When the in silico prediction tool was applied to the variant types, all results revealed the potential harmfulness of these variants with respect to function. Based on these results and with consideration for changes in amino acids, variant frequency, segregation analysis, and the occurrence of variants *in trans*, four variants (c.741C > A, c.867C > A, and c.1037‐2A > T of *TYR*, and c.695dupT of *OCA2*) were classified as pathogenic. The remaining variants (c.731G > A of *TYR*, c.1054A > G of *OCA2*, and c.9C > A of *HPS1*) were classified as likely pathogenic.

**TABLE 3:** Pathogenicity analysis of novel variants

Gene	Variant	Type	Minor allele frequency (TOPMed/gnomAD/ExAC/1000G)	Pathogenicity prediction (SIFT/PolyPhen‐2/Provean/MutationTaster/HSF)	Evidence criterion	Classification
*TYR*	c.731G > A (p.C244Y)	Missense	0.000008/—/—/—	Damaging/Probably Damaging/Deleterious/disease causing/—	PM2+PM3+PP1+PP3+PP4	Likely pathogenic
	c.741C > A (p.C247*)	Nonsense	—/0.000008/—/—	—/—/—/disease causing/—	PVS1+PM2+PP4	Pathogenic
	c.867C > A (p.C289*)	Nonsense	—/—/—/—	—/—/—/disease causing/—	PVS1+PM2+PM3+PP4	Pathogenic
	c.1037‐2A > T	Splicing	—/—/—/—	—/—/—/disease causing/most probably affecting splicing	PVS1+PM2+PM3+PP1+PP4	Pathogenic
*OCA2*	c.695dupT (p.A233Gfs*26)	Frameshift	—/—/—/—	—/—/—/disease causing/—	PVS1+PM2+PM3+PP4	Pathogenic
	c.1054A > G (p.R352G)	Missense	—/—/—/—	Damaging/Probably Damaging/Deleterious/disease causing/—	PM2+PM3+PP3+PP4	Likely pathogenic
*HPS1*	c.9C > A (p.C3*)	Nonsense	—/—/—/—	—/—/—/disease causing/—	PVS1+PM2	Likely pathogenic

### 3.4 Prenatal genetic diagnosis

Among the 20 families, six pregnant women (family 2, 3, 5, 7, 17, and 18) underwent amniocentesis for prenatal diagnosis. Genetic tests on six fetuses revealed three carrier fetuses, two normal fetuses, and one affected fetus. The fetus of family 5 carried compound heterozygous variants (c.832C > T and c.896G > A) in the *TYR* gene, the same result as identified in the proband; this fetus was diagnosed as affected. The fetuses of family 3 and family 18 inherited the normal allele from the carrier parent and, hence, were diagnosed as normal. Only one variant in *TYR* was identified in three fetuses, including the fetus of family 2 which was heterozygous for the c.229C > T variant, the fetus of family 7 which was heterozygous for the c.832C > T variant, and the fetus of family 17 which was heterozygous for the c.230_232dupGGG variant. Maternal contamination was excluded from all prenatal diagnostic samples by 16 STR loci analysis. Following further genetic counseling, the parents of fetus 5 elected to terminate the pregnancy, while the remaining couples decided to continue their pregnancies. The children all presented with a normal appearance at birth; thus, the follow‐up results were consistent with the results of prenatal diagnosis.

## 4 DISCUSSION

This study successfully identified the genotypes of 20 OCA patients by Sanger sequencing and WES. It should be noted that Sanger sequencing performed on family 19 and family 20 failed to identify any pathogenic variations due to the atypical phenotypes, while subsequent WES revealed pathogenic variants in *OCA2* in family 19 and *HPS1* in family 20. This indicates that new techniques such as WES can help to identify causal variants in rare or atypical subtypes of OCA, which are likely to be missed by Sanger sequencing.

The distribution of OCA genes and the variant alleles of these genes vary according to ethnicity. *TYR* is the most common OCA gene throughout the world. Sequence variants in the *OCA2* gene have been described in all major ethnic groups and are most common in African and African American populations.^10^ In contrast, *TYRP1* variants and *HPS1* variants are prevalent in Southern Africa and Northwestern Puerto Rico, respectively.^11^, ^12^ In this study of Chinese OCA patients, variants in *TYR*, *OCA2*, and *HPS1* were found in 85%, 10%, and 5%, of patients, respectively, which is similar to the report of Wei et al^13^ In contrast, *SLC45A2* variants were totally absent here but have been reported to account for 10%‐20% of Chinese OCA cases.^13^, ^14^ This highlights the genetic heterogeneity of OCA even in patients from the same ethnic group. In addition, differences in the included number of cases may be another explanation.

Sixteen distinct variants in the *TYR* gene were identified in this study. Aside from one splice site variant (c.1037‐2A > T) in the junction region of exon 3 and intron 2, all other variants were located in exon 1, 2, and 4. Among these, variants in exon 1 and 2 collectively accounted for 81% (13/16), highlighting the hotspot regions of *TYR* variants. This finding is consistent with a previous study.^13^ Of the 34 *TYR* gene variant alleles, c.895C > A (p.R299S) and c.896G > A (p.R299H), both of which changed codon 299 in the CuA region, were the most common variations in our study, with each identified in six probands (17.6%, 6/34). The latter is a known hotspot variant and the former is common in Chinese OCA patients as well.^13^, ^15^

More than 400 pathogenic variants in *TYR* have been identified to date, and here, we identified four additional novel variants in five families. The c.731G > A variant was detected in two unrelated patients (patient 6 and 8) diagnosed as OCA type 1. This variant caused cysteine to be substituted by tyrosine at codon 244, showing a high level of sequence conservation; thus, the p.C244Y variant may have deleterious effects on tyrosinase. c.741C > A (p.C247*) in patient 13 and c.867C > A (p.C289*) in patient 3 resulted in a truncated protein and led to a loss of tyrosinase activity. These two nonsense variants are reported here for the first time, while missense variants on the same amino acid, p.C247R, p.C289R, p.C289G, and p.C289Y, have been previously reported.^16^, ^17^, ^18^, ^19^ Patient 9 was found to have compound heterozygosity of the novel c.1037‐2A > T and the known pathogenic variant c.346C > T (p.R116*). Through segregation analysis, these two variants were also identified in his affected brother who exhibited a similar phenotype, while their normal sister carried the single variant c.1037‐2A > T. The splicing variant c.1037‐2A > T was predicted to most likely affect the splicing pattern of exons.

*OCA2* (previously called *P*) is the only causal gene implicated in OCA type 2; about 300 variants implicated in *OCA2* have been reported to date. In this study, patient 18 and 19 were both compound heterozygotes, with each possessing a known variant and a novel variant, c.695dupT (p.A233Gfs*26) and c.1054A > G (p.R352G), respectively. The frameshift c.695dupT introduces a 1‐bp duplication in exon 7 of *OCA*2, resulting in a frameshift distal to codon 233 with the termination of the nonsense polypeptide at codon 258. Several nonsense variants downstream of codon 258 have been reported to be pathogenic^13^, ^20^; thus, c.695dupT (p.A233Gfs*26) is suggested to be a loss‐of‐function variant and might lead to inactivity of the P protein. The c.1054A > G (p.R352G) variant of *OCA2* in patient 19 caused arginine to be substituted by glycine at codon 352 and was predicted to be deleterious by all in silico tools. Patient 19 also carried another known pathogenic variant, c.1426A > G (p.N476D).^21^ We further determined that the c.1054A > G variant was *in trans* (different copy of *OCA2* gene) with the known variant by testing the next generation of the patient.

HPS is a rare genetic disorder characterized by oculocutaneous albinism, bleeding tendency and, in some cases, lysosomal storage disease.^22^, ^23^ As of April 2019, 68 variants in *HPS1* were listed in the HGMD. Here, we found patient 20 was homozygous for a novel nonsense variant c.9C > A (p.C3*) in the *HPS1* gene. Compared with various *HPS1* variants in the known databases, including the ClinVar, HGMD, and Albinism databases, this variant is predicted to cause the shortest truncated protein to lose most of the wild‐type *HPS1* sequence, keeping only three amino acid residues. The female patient with this novel variant showed obvious hypopigmentation and characteristic ophthalmological findings, but no hemorrhagic problems; all coagulation tests and routine blood tests were normal. Similar characteristics were reported in two *HPS1* patients in the study by Luo et al^24^ and one female patient in the study by Wei et al^25^; these three patients were described as compound heterozygote for p.E63* and p.Q603*, compound heterozygote for c.399‐14G > A and p.M325Hfs*128, and homozygous for p.D644Dfs*79, respectively. The phenotypes of these four patients were different from the majority of HPS individuals who experience reduced platelet aggregation abilities besides OCA.^26^ As Oh et al^27^ suggested, differentially truncated HPS1 polypeptides may have different consequences for subcellular function. The c.9C > A (p.C3*) variant, reflecting truncated alleles located near the N‐terminus, might result in a serious OCA phenotype of HPS but no clear hemorrhagic diathesis. Further studies on the function of this variant will reveal the underlying mechanism.

In summary, this study successfully detected causal variants in 20 OCA families. A total of 21 distinct variants of the *TYR*, *OCA2*, and *HPS1* genes were identified by direct Sanger sequence and WES, of which, seven variants were novel. In addition, effective prenatal diagnosis and genetic counseling were provided to six OCA families to assist them to avoid the birth of affected children. This study expands our understanding of the genotypic spectrum of the Chinese OCA population and highlights that the development of new genetic testing technologies can contribute to more accurate and efficient clinical diagnoses.

## AUTHORS' CONTRIBUTIONS

All authors contributed to the study conception and design. Chenyang Xu and YanBao Xiang designed the study, interpreted the clinical data, and wrote the article. Huanzheng Li, Yunzhi Xu, Xueqin Xu, and Yijian Mao collected samples, genotyped the cases, and finished the follow‐up. Lili Zhou and Shaohua Tang performed genetic counseling. Chenyang Xu and Shaohua Tang helped in the statistical analysis. All authors read and approved the final manuscript.

## Supporting information

Table S1‐S2

## Data Availability

The data that provided the evidence for the study are available from the corresponding author upon reasonable request.
