# Supplementary material for: Genetic analysis and prenatal diagnosis of 20 Chinese families with oculocutaneous albinism
Source: J Clin Lab Anal. 2020 Oct 30;35(2):e23647. doi: 10.1002/jcla.23647 (PMC7891544; doi:10.1002/jcla.23647)
Supplement: Supplementary file 1 — Table S1‐S2 [file JCLA-35-e23647-s001.docx]

TABLE S1 Primers used in PCR for amplification of *TYR*

| Exon | Forward Primer | Reverse Primer | Product size（bp） |
| --- | --- | --- | --- |
| 1-A | CAGTTCCTGCAGACCTTGTG | CTGGGGCACTCAAATCGAAG | 423 |
| 1-B | CCAAACTGCACAGAGAGACG | CCTCCCTACTCTGACATCGT | 530 |
| 2 | CTGACTCAGTGGTGGTGAC | CCAGTGTCAGCTAGGGTCAT | 426 |
| 3 | AGGCAGAATGAACAGGAGGG | AGGCACCCTCTATTTAAATCCAA | 483 |
| 4 | ATGTTTCTTAGTCTGAATAACC | ACTAGATTCAGCAATTCCTCT | 257 |
| 5 | CTCCAAAGGACTGTGAAAGG | CTGGGAACCTGGACATTACT | 383 |

TABLE S2 33 candidate genes responsible for albinism or diseases whose phenotypes partially overlap with albinism

| NO. | Gene | NO. | Gene | NO. | Gene |
| --- | --- | --- | --- | --- | --- |
| 1 | *ADFN* | 12 | *HERC2* | 23 | *OASD* |
| 2 | *AP3B1* | 13 | *HPS1* | 24 | *OCA2* |
| 3 | *BLOC1S3* | 14 | *HPS3* | 25 | *OCA5* |
| 4 | *BLOC1S6* | 15 | *HPS4* | 26 | *PAX6* |
| 5 | *C10orf11* | 16 | *HPS5* | 27 | *RAB27A* |
| 6 | *CACNA1F* | 17 | *HPS6* | 28 | *SLC24A5* |
| 7 | *DTNBP1* | 18 | *KIT* | 29 | *SLC38A8* |
| 8 | *EDNRB* | 19 | *LYST* | 30 | *SLC45A2* |
| 9 | *EPG5* | 20 | *MC1R* | 31 | *SNAI2* |
| 10 | *FHASD* | 21 | *MITF* | 32 | *TYR* |
| 11 | *GPR143* | 22 | *MYO5A* | 33 | *TYRP1* |
